# Copy Number Variations in the *Survival Motor Neuron* Genes: Implications for Spinal Muscular Atrophy and Other Neurodegenerative Diseases

**DOI:** 10.3389/fmolb.2016.00007

**Published:** 2016-03-10

**Authors:** Matthew E. R. Butchbach

**Affiliations:** ^1^Center for Applied Clinical Genomics, Nemours Biomedical Research, Nemours Alfred I. duPont Hospital for Children Wilmington, DE, USA; ^2^Center for Pediatric Research, Nemours Biomedical Research, Nemours Alfred I. duPont Hospital for Children Wilmington, DE, USA; ^3^Department of Biological Sciences, University of Delaware Newark, DE, USA; ^4^Department of Pediatrics, Thomas Jefferson University Philadelphia, PA, USA

**Keywords:** spinal muscular atrophy, amyotrophic lateral sclerosis, progressive muscular atrophy, neurodegenerative disease, copy number variation, *SMN1*, *SMN2*

## Abstract

Proximal spinal muscular atrophy (SMA), a leading genetic cause of infant death worldwide, is an early-onset, autosomal recessive neurodegenerative disease characterized by the loss of spinal α-motor neurons. This loss of α-motor neurons is associated with muscle weakness and atrophy. SMA can be classified into five clinical grades based on age of onset and severity of the disease. Regardless of clinical grade, proximal SMA results from the loss or mutation of *SMN1* (*survival motor neuron 1*) on chromosome 5q13. In humans a large tandem chromosomal duplication has lead to a second copy of the *SMN* gene locus known as *SMN2*. *SMN2* is distinguishable from *SMN1* by a single nucleotide difference that disrupts an exonic splice enhancer in exon 7. As a result, most of *SMN2* mRNAs lack exon 7 (*SMN*Δ*7*) and produce a protein that is both unstable and less than fully functional. Although only 10–20% of the *SMN2* gene product is fully functional, increased genomic copies of *SMN2* inversely correlates with disease severity among individuals with SMA. Because *SMN2* copy number influences disease severity in SMA, there is prognostic value in accurate measurement of *SMN2* copy number from patients being evaluated for SMA. This prognostic value is especially important given that *SMN2* copy number is now being used as an inclusion criterion for SMA clinical trials. In addition to SMA, copy number variations (CNVs) in the *SMN* genes can affect the clinical severity of other neurological disorders including amyotrophic lateral sclerosis (ALS) and progressive muscular atrophy (PMA). This review will discuss how *SMN1* and *SMN2* CNVs are detected and why accurate measurement of *SMN1* and *SMN2* copy numbers is relevant for SMA and other neurodegenerative diseases.

## Introduction

Proximal spinal muscular atrophy (SMA) is a leading genetic cause of infant death worldwide, alongside cystic fibrosis. The incidence of SMA is 1 in 6000–10,000 live births (Pearn, 1978; Cuscó et al., 2002; Sugarman et al., 2012). The carrier frequency for SMA is 1:25–50 in most populations (Ben-Shachar et al., 2011; Su et al., 2011; Lyahyai et al., 2012; Sugarman et al., 2012) though it is lower for some ethnicities (Zaldívar et al., 2005; Labrum et al., 2007; Hendrickson et al., 2009; Sangaré et al., 2014).

SMA is an early-onset neurodegenerative disease characterized by the loss of α-motor neurons in the anterior horn of the spinal cord, i.e., lower motor neurons (LMNs; Crawford and Pardo, 1996; Kolb and Kissel, 2015). This loss of α-motor neurons is associated with muscle weakness and atrophy. In SMA, those muscles that are proximally innervated are preferentially affected over distal muscles. SMA can be classified into five clinical grades based on age of onset and severity of the disease (Munsat and Davies, 1992; Russman, 2007; Table 1). Type 0 SMA infants present with very severe hypotonia and require respiratory support from birth. These SMA infants cannot survive beyond 6 months. Type I SMA [listed in the Online Inheritance in Man (OMIM) database under accession number 253300; http://www.omim.org/entry/253300] patients have an age of onset before 6 months. They show hypotonia and weakness in limbs; they are unable to sit independently. Type I SMA infants show a bell-shaped chest due to weakness in the intercostals muscles but sparing of the diaphragm; this bell-shaped chest results in abnormal breathing patterns. These patients typically live < 2 years. Type II SMA (http://www.omim.org/entry/253500) patients have an age of onset before 18 months. They are poor crawlers and weak sitters; most of these patients can rarely stand and only with support. Their legs are generally weaker than their arms. Due in part to better supportive care, these patients generally have a life expectancy into early adulthood. Type III SMA (http://www.omim.org/entry/253400) patients have an age of onset >18 months. These patients are able to walk with difficulty (waddling gait) and the legs are weaker than the arms. Type III patients usually have a normal lifespan. Adult-onset (type IV) SMA (http://www.omim.org/entry/271150) patients have an age of onset of 18–21 years. Type IV SMA exhibits as a slowly progressive limb weakness. The disease is fairly benign in these patients.

**Table 1:** **Clinical classification of spinal muscular atrophy (SMA)**.

**Type**	**Age of onset**	**Requires respiratory support at birth**	**Able to sit**	**Able to stand**	**Able to walk**	**Life expectancy**	**Predicted *SMN2* copy number**
0	Prenatal	Yes	No	No	No	<6 months	1
1	<6 months	No	No	No	No	<2 years	2
2	6–18 months	No	Yes	No	No	10–40 years	3
3	>18 months	No	Yes	Yes	Assisted	Adult	3–4
4	>5 years	No	Yes	Yes	Yes	Adult	>4

## Genetics of SMA

**Figure 1:**
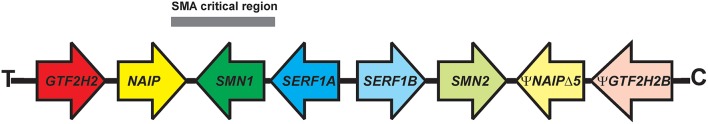
**Organization of the inverted duplication locus on 5q13**. 4 protein-coding genes—*GTF2H2* (*general transcription factor IIH*), *NAIP* (*neuronal apoptosis inhibitory protein*), *SMN1* (*survival motor neuron 1*), and *SERF1A* (*small EDRK-rich factor 1A*)—are present within the 500-kilobase inverted duplication on the long arm of chromosome 5 (5q13.2). The duplicated genes are Ψ*GTF2H2B* (*GTF2H2 pseudogene*), Ψ*NAIP*Δ*5* (*NAIP pseudogene with loss of exon 5*), *SMN2*, and *SERF1B*. The SMA critical region is under the gray bar. C, centromeric end; T, telomeric end.

SMA is an autosomal recessive disorder (Brandt, 1949). Linkage analysis (Brzustowicz et al., 1990; Gilliam et al., 1990; Melki et al., 1990a,b) along with genetic and physical mapping studies (reviewed in Morrison, 1996) identified the SMA locus on the long arm of chromosome 5, specifically in the 5q13 region. There is a 500 kilobase (kb) inverted segmental duplication within this region of chromosome 5 that is unique to humans (Courseaux et al., 2003; Schmutz et al., 2004). Four protein-coding genes have been identified within this region (Figure 1): *SMN1* [*survival motor neuron 1, telomeric SMN* (*SMN*^*T*^; Lefebvre et al., 1995)], *NAIP* [*neuronal apoptosis inhibitor protein* (Roy et al., 1995)], *GTF2H2A* [*general transcription factor IIH, p44* (Bürglen et al., 1997; Carter et al., 1997)], and *SERF1A* [*small EDRK-rich factor 1A, H4F5A* (Scharf et al., 1998)]. The duplicated genes are either identical to their partner gene (*SERF1B*), different in a small number of nucleotides [*SMN2* or *centromeric SMN* (*SMN*^*C*^)] or are pseudogenes (Ψ*GTF2H2B* and Ψ*NAIP*Δ*5*).

In more than 95% of cases, proximal SMA results from the loss of *SMN1* but retention of *SMN2*, regardless of clinical grade (Lefebvre et al., 1995). Large-scale deletions in chromosome 5q13 that include *SMN1, NAIP, SERF1A*, and *GTF2H2A* are observed in patients with type I SMA (Wirth et al., 1995; Burlet et al., 1996; Rodrigues et al., 1996; Velasco et al., 1996; Bürglen et al., 1997; Carter et al., 1997). Smaller deletions only involving *SMN1* have also been observed in type I SMA patients demonstrating that *SMN1* is the most likely causative gene for SMA. In addition, the identification of intragenic SMA mutations in *SMN1* (Lefebvre et al., 1995; see Burghes and Beattie, 2009 for a comprehensive listing of SMA-associated point mutations in *SMN1*) provides additional evidence to support *SMN1* as the gene responsible for SMA. To date, no intragenic mutations in the other genes within this segmental duplication have been associated with SMA.

**Figure 2:**
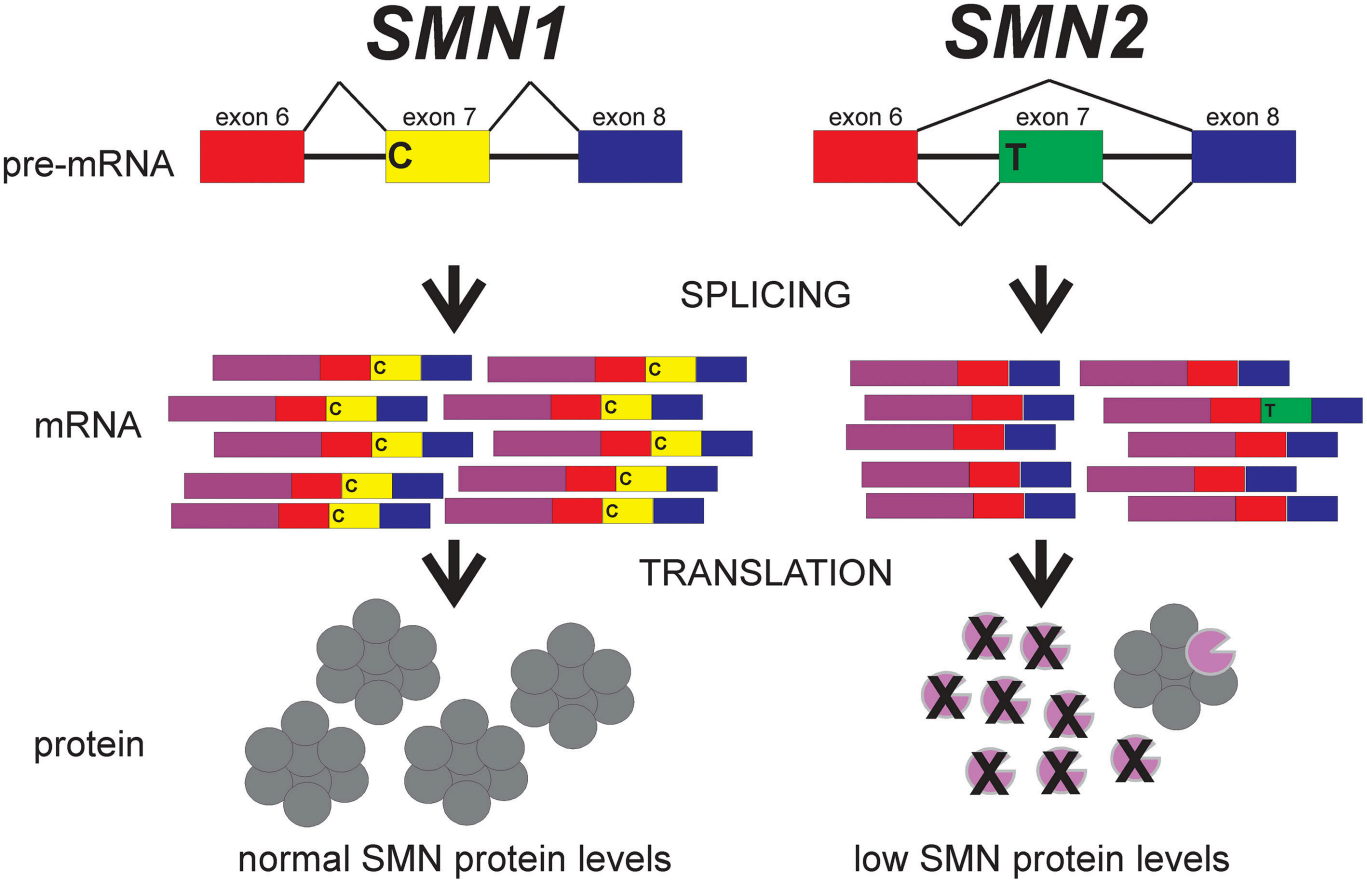
**The effect of the C-to-T transition in exon 7 between ***SMN1*** and ***SMN2*** on splicing**. Adapted from Butchbach and Burghes (2004).

As mentioned earlier, the *SMN* gene is duplicated in humans to give rise to *SMN1* and *SMN2*. This duplication of *SMN* is unique to humans (Rochette et al., 2001). What is the difference between *SMN1* and *SMN2*? The major difference between these two *SMN* genes is a C-to-T transition in exon 7(*SMN2 c.850C*>*T*; Lorson et al., 1999; Monani et al., 1999). This nucleotide change is translationally silent. This position on exon 7 is in the middle of an exonic splicing enhancer (ESS) sequence that regulated the inclusion of exon 7 in SMN transcripts (Figure 2). For *SMN1*, the C at this position promotes inclusion of exon 7 in *SMN1*-derived mRNAs which leads to the production of full-length SMN protein. Full-length SMN protein is able to form functional complexes. For *SMN2*, the T at this position disrupts this ESS which results in the exclusion of exon 7 (SMNΔ7) from the majority of *SMN2*-derived mRNAs. As a result, a truncated SMNΔ7 protein is produced by the majority (~90%) of *SMN2*-derived mRNAs; this SMNΔ7 protein is unstable and is unable to associate with itself (Lorson and Androphy, 2000; Burnett et al., 2009; Cho and Dreyfuss, 2010). The SMNΔ7 protein is still partially functional given that transgenic overexpression of SMNΔ7 in severe SMA mice partially ameliorates their phenotype since these mice die at 14–15 days as opposed to 5–8 days (Le et al., 2005). About 10% of the mRNAs from *SMN2* contain exon 7 and these full-length mRNAs can produce some full-length, functional SMN protein.

## Biology of SMN

SMN is a ubiquitously expressed protein whose expression is reduced in SMA. There is a strong inverse correlation between SMN protein levels and disease severity in SMA fibroblasts and lymphoblastoid cells as well (Coovert et al., 1997; Lefebvre et al., 1997; Kolb et al., 2006). Changes in SMN mRNA and protein levels observed in SMA patient-derived PBMCs mirror those observed in SMA cell lines (Sumner et al., 2006; Simard et al., 2007; Vezain et al., 2007; Tiziano et al., 2010; Crawford et al., 2012). SMN protein is present within the nuclei in discreet foci known as gems (Liu and Dreyfuss, 1996). In SMA fibroblasts, the number of SMN-positive subnuclear gems is higher in cells derived from mild SMA individuals than in those from children with more severe forms of SMA (Coovert et al., 1997).

SMN is required for the assembly of the small nuclear ribonucleoprotein (snRNP) complexes that mediate splicing (Pellizzoni, 2007; Burghes and Beattie, 2009). snRNP assembly is defective in SMN-deficient SMA cells (Wan et al., 2005). Since snRNP assembly is required for all cell types, why are motor neurons primarily affected in SMA? snRNP assembly is defective in tissues from mouse models for SMA and that the extent of reduced snRNP assembly correlates with phenotypic severity of these SMA mice (Gabanella et al., 2005; Zhang et al., 2008). snRNP assembly is more markedly reduced in SMA mouse neural tissues than in other tissues like the kidney (Gabanella et al., 2005) suggesting that motor neurons are more sensitive to deficits in snRNP assembly. SMN may also have a function that is unique to motor neurons. Axonal defects in *Smn*-knocked down zebrafish embryos (McWhorter et al., 2003) are corrected by overexpression of mutant SMNs which are incapable of snRNP assembly (Carrel et al., 2006).

## *SMN2* CNV in SMA

The number of *SMN2* copies in the genome varies between 0 and 8. Numerous studies have demonstrated an inverse relationship between *SMN2* copy number and disease severity among in SMA (Lefebvre et al., 1995, 1997; van der Steege et al., 1995; Coovert et al., 1997; McAndrew et al., 1997; Taylor et al., 1998; Feldkötter et al., 2002; Mailman et al., 2002; Anhuf et al., 2003; Gérard et al., 2004; Prior et al., 2005; Su et al., 2005; Swoboda et al., 2005; Arkblad et al., 2006; Scarciolla et al., 2006; Wirth et al., 2006; Gómez-Curet et al., 2007; Huang et al., 2007; Tiziano et al., 2007; Elsheikh et al., 2009; Wang et al., 2010a,b, 2014a; Alías et al., 2011; Amara et al., 2012; Crawford et al., 2012; Dobrowolski et al., 2012; Kirwin et al., 2013; Qu et al., 2014; Brkušanin et al., 2015; Fang et al., 2015; Stabley et al., 2015). Patients with milder forms of SMA have higher *SMN2* copy numbers than severe SMA patients (Table 1).

Mice have only 1 *SMN* gene, *mSmn*, which is orthologous to *SMN1* (DiDonato et al., 1997a; Viollet et al., 1997). Loss of *mSmn* leads to embryonic lethality (Schrank et al., 1997). Conditional loss of *mSmn* in specific cell types like neurons, myofibers and hepatocytes results in loss of those cells *in vivo* (Cifuentes-Diaz et al., 2002; Nicole et al., 2003; Vitte et al., 2004). Transgenic insertion of *SMN2* rescues the embryonic lethality observed in *mSmn* nullizygous mice (Hsieh-Li et al., 2000; Monani et al., 2000; Michaud et al., 2010). While two copies of *SMN2* rescues embryonic lethality in *mSmn*-deficient mice, these mice develop a very severe motor phenotype and die within 8 days after birth (Hsieh-Li et al., 2000; Monani et al., 2000). Those *mSmn*-deficient mice with 3-4 *SMN2* copies exhibit a milder SMA phenotype than the two copy *SMN2* SMA mice (Hsieh-Li et al., 2000; Michaud et al., 2010). If the *SMN2* copy number is high (i.e., 8), then the resultant *mSmn*-deficient mice exhibit no signs of SMA and are phenotypically normal (Monani et al., 2000). *SMN2* CNV, therefore, is a major modifier of disease severity in SMA in mice as well as in humans.

Gene conversion is one mechanism to account for increased *SMN2* copy number in the absence of *SMN1* in SMA (Burghes, 1997). In this scenario, the *SMN1* gene actually contains part of *SMN2*, in particular within exon 7 (Wirth et al., 1995; Devriendt et al., 1996; Hahnen et al., 1996; van der Steege et al., 1996; Campbell et al., 1997; DiDonato et al., 1997b). It is hypothesized that type I SMA patients have deletions of *SMN1* on both chromosomes. Type II SMA patients have an *SMN1* deletion on one chromosome and a *SMN1*-to-*SMN2* conversion on the other chromosome (three copies of *SMN2*). Type III SMA patients have *SMN1*-to-*SMN2* converted genes on both chromosomes (four copies).

While the inverse relationship between *SMN2* copy number and disease severity generally holds true in SMA, there are some exceptions. For example, there are cases of type II and III SMA patients who harbor only two copies of *SMN2* instead of the predicted three or four copies (Prior et al., 2009; Bernal et al., 2010; Vezain et al., 2010). Sequencing of *SMN2* in these cases revealed the presence of a rare single nucleotide variant (*SMN2 c.859G*>*C*) in exon 7 (Prior et al., 2009; Bernal et al., 2010; Vezain et al., 2010). This variant regulates the splicing of *SMN2* pre-mRNAs so that a greater proportion of *SMN2* transcripts contain exon 7. This variant may either create an additional SF2/ASF binding to promote exon 7 inclusion (Prior et al., 2009) or disrupt a hnRNPA1-dependent splicing silencer element (Vezain et al., 2010).

Intrafamilial variability in clinical presentation has been reported in SMA families with more than one affected sibling (Burghes et al., 1994; Cobben et al., 1995; Hahnen et al., 1995; McAndrew et al., 1997; Cuscó et al., 2006). Even though the siblings are haploidentical with respect to *SMN2* copy number, they have differing clinical presentations. This would suggest that there are *SMN2*-independent modifiers of disease severity for SMA. *Plastin-3* (*PLS3*) mRNA levels were higher in females with milder SMA than in discordant siblings with a more severe SMA clinical presentation (Oprea et al., 2008; Stratigopoulos et al., 2010; Bernal et al., 2011; Yanyan et al., 2014). In some families, however, female siblings with a more severe SMA phenotype show high *PLS3* mRNA levels (Bernal et al., 2011). It is possible that the modifier property of *PLS3* is age- and sex-dependent as well as incompletely penetrant; alternatively, *PLS3* may not actually be a major modifier of SMA phenotype. There may be other non-*SMN2* molecular modifiers of disease severity in SMA. It is important to identify and characterize these novel modifiers for the development of novel SMA biomarkers and targets for the development of therapeutic strategies for SMA as well as for the planning of current and future clinical trials in SMA (Wirth et al., 2013).

## Measuring *SMN1* and *SMN2* CNVs

Because *SMN2* copy number influences disease severity in SMA, there is prognostic value in accurate measurement of *SMN2* copy number from patients being evaluated for SMA. Molecular diagnosis of SMA— i.e., loss of *SMN1*—has historically been made using a polymerase chain reaction (PCR)-based assay followed by digestion of the PCR product with specific restriction endonucleases (PCR-RFLP; Lefebvre et al., 1995; van der Steege et al., 1995). Numerous assays have since been developed to quantify *SMN2* copy number in DNA samples from SMA patients. These assays include radioactive PCR (Coovert et al., 1997; McAndrew et al., 1997), fluorescent PCR (Taylor et al., 1998), quantitative (real-time) PCR (qPCR; Feldkötter et al., 2002; Anhuf et al., 2003; Gómez-Curet et al., 2007), competitive PCR/primer extension (Gérard et al., 2004), denaturing high performance liquid chromatography (Su et al., 2005), multiplex ligation-dependent probe amplification (MLPA; Arkblad et al., 2006; Scarciolla et al., 2006; Huang et al., 2007; Alías et al., 2011; Fang et al., 2015), quantitative capillary electrophoresis fragment analysis (Kirwin et al., 2013), short-amplicon melt profiling (Dobrowolski et al., 2012), fluorescent multiplex PCR/capillary electrophoresis (Wang et al., 2010a,b), and universal fluorescent triprobe ligation (Wang et al., 2014a). An important limitation of these established PCR-based copy number assays is the requirement for a parallel-run calibration curve to assign a breakpoint necessary that identifies placement of an ordinal *SMN2* value. Additionally, these techniques cannot easily distinguish unit differences in *SMN1* or *SMN2* when the copy number is >3 (Gómez-Curet et al., 2007; Alías et al., 2011; Prior et al., 2011).

To overcome some of the limitations associated with the PCR-based assays described above, digital PCR (dPCR) distributed across a large number of partitions by limited dilution so that some partitions will lack the template DNA (Sykes et al., 1992; Vogelstein and Kinzler, 1999). The absolute abundance of the target gene can be measured by counting the number of positive partitions and the number of negative partitions. dPCR can reliably and accurately measure *SMN1* and *SMN2* copy numbers over a wide range, i.e., between 0 and 6 copies (Zhong et al., 2011; Stabley et al., 2015).

## *SMN1* and *SMN2* CNVs in ALS

Amyotrophic lateral sclerosis (ALS) is a mostly adult-onset motor neuron disease characterized by a progressive loss of motor function leading to paralysis and respiratory failure (Boylan, 2015; Statland et al., 2015). Unlike SMA, ALS is caused by degeneration of LMNs as well as upper motor neurons (UMNs). ALS is usually fatal within 3–5 years after disease onset but there is considerable variability with respect to duration as well as phenotypic presentation (Swinnen and Robberecht, 2014). Most cases of ALS are sporadic in nature since there is no apparent family history. Approximately 10% of ALS is considered familial since either a causative gene has been identified or there is strong family history. With the recent advents of whole exome and whole genome sequencing, the genetic bases of almost 70% of familial ALS and 10% of sporadic ALS have been identified (Renton et al., 2014).

There are many case studies reporting the co-occurrence of SMA and ALS within a family (Appelbaum et al., 1992; Camu and Billiard, 1993; Orrell et al., 1997; Corcia et al., 2002a) which suggests that *SMN1* deficiency may lead to ALS in addition to SMA. *SMN1* deletions, however, have not been observed in either familial or sporadic ALS patients (Orrell et al., 1997; Corcia et al., 2002a). Furthermore, no intragenic point mutations in *SMN1* have been reported in the ALS population (Blauw et al., 2012). The intrafamilial coexistence of SMA and ALS, therefore, occurs by chance.

Even though loss of *SMN1* is not associated with ALS, CNVs in the *SMN* genes may modulate the clinical severity of ALS in addition to SMA. Multiple studies suggest that deletion of *SMN2* leads to increased risk of the sporadic forms of amyotrophic lateral sclerosis (ALS) (Veldink et al., 2001, 2005; Kim et al., 2010; Corcia et al., 2012; Lee et al., 2012). Additionally, atypical *SMN1* copy number—in other words, any number other than two—can affect the risk of ALS (Corcia et al., 2002b, 2006; Blauw et al., 2012; Wang et al., 2014b). Other studies, however, have shown no association between deletion of either *SMN1* or *SMN2* in ALS (Jackson et al., 1996; Moulard et al., 1998; Parboosingh et al., 1999; Crawford and Skolasky, 2002; Gamez et al., 2002). The discrepant results from these studies may be due, in part, to different assays used to assess *SMN1* and *SMN2* CNVs as some reports using quantitative PCR while others used MLPA or RFLP.

SMN and some ALS-associated proteins are involved in common biochemical pathways. Both familial and sporadic ALS have been linked to mutations in *fused in sarcoma* (*FUS)* (Kwiatkowski et al., 2009; Vance et al., 2009) (OMIM #608030) as well as in *TAR DNA binding protein-43 kDa* (*TDP-43*) (Kabashi et al., 2008; Sreedharan et al., 2008) (OMIM #612069). Both FUS and TDP-43 colocalize with SMN in subnuclear gems and ALS-associated mutations in *FUS* and *TDP-43* reduce gem localization of SMN (Shan et al., 2010; Yamazaki et al., 2012; Gerbino et al., 2013; Groen et al., 2013; Ishihara et al., 2013; Sun et al., 2015). Gem localization of SMN, however, is not altered in other forms of sporadic ALS (Kariya et al., 2014). These mutant proteins also disrupt the SMN-mediated assembly of the splicing machinery by disrupting the interaction between SMN and U1-snRNPs (small nuclear ribonucleoprotein particles; Gerbino et al., 2013; Tsuiji et al., 2013; Sun et al., 2015; Yu et al., 2015). Additionally, ALS-associated FUS mutations disrupt the localization of SMN to axons (Groen et al., 2013). The SMN function, therefore, may be disrupted in certain forms of ALS.

Ectopic overexpression of SMN protects NSC34 motor neuron-like cells from cell death induced by ALS-associated mutant *superoxide dismutase 1* (*SOD1*) (OMIM #105400) (Zou et al., 2007). The SOD1(G93A) transgenic mouse model for ALS that also harbors a knockout of 1 *mSmn* allele (*SOD1(G93A)*^+∕−^*;mSmn*^+∕−^) exhibits a more severe ALS phenotype than SOD1(G93A) ALS mice (*SOD1(G93A)*^+∕−^*;mSmn*^+∕+^) (Turner et al., 2009). Furthermore, ectopic overexpression of SMN in neurons and glia improves motor function of and delays motor neuron loss in SOD1(G93A) ALS mice (Turner et al., 2014). Strong transgenic overexpression of *SMN2*—in other words, eight copies of *SMN2*—delayed disease onset in the SOD1(G86R) mouse model for ALS (Kariya et al., 2012). Thesestudies suggest that increasing SMN expression may modulate disease severity in ALS. It will be interesting to determine the effect of *SMN2* overexpression on disease severity in FUS- and TDP-43-associated ALS.

## *SMN1* and *SMN2* CNVs in PMA

Progressive muscular atrophy (PMA) is an adult-onset motor neuron disease characterized by loss of LMNs (Rowland, 2010; Liewluck and Saperstein, 2015). It is a rare and sporadic disorder that is clinically distinct from ALS even though subclinical involvement of UMNs has been observed in many PMA patients. Those PMA patients exhibiting a more severe clinical presentation tend to harbor higher *SMN1* copy numbers (Kuzma-Kozakiewicz et al., 2013). No relationship between *SMN2* copy number and disease severity was noted in these PMA patients. Moulard et al. (1998) noted that the frequency of *SMN2* deletion was higher in a small group of patients with sporadic LMN disease.

## Conclusions

CNVs in the *SMN* genes can modulate disease severity in SMA as well as in other motor neurons. It is well-established that *SMN2* copy number is inversely correlated with disease severity in SMA. Because of this relationship, *SMN2* is a primary target for the development of therapeutics for SMA (Arnold and Burghes, 2013; Cherry et al., 2014). Numerous approaches including promoter activation, increased inclusion of exon 7 and protein stabilization are currently being developed to increase *SMN2* expression. With respect to other motor neuron diseases, it is presently unclear whether increasing *SMN1* or *SMN2* expression would be beneficial or detrimental. On one hand, increasing SMN expression provides neuroprotective benefit to cell culture and transgenic mouse models for ALS; however, some genetic studies suggest that duplication of *SMN1* increases the risk of sporadic ALS. Future studies will assess the relationship between *SMN1* and *SMN2* CNVs and disease risk and progression in ALS and PMA as well as in other disorders affecting motor neurons.

*SMN2* copy number is becoming an inclusion criterion for many clinical trials for SMA. Additionally, *SMN2* copy number can be used to help guide the type of care SMA patients will receive. It is, therefore, essential to be able to accurately and reliably measure *SMN2* CNVs in SMA patient samples, especially for those individuals harboring more than three copies of *SMN2*. Newly developed technologies like dPCR offer a means to accurately determine *SMN2* copy number over a wider range.

## Author contributions

The author confirms being the sole contributor of this work and approved it for publication.

### Conflict of interest statement

The author declares that the research was conducted in the absence of any commercial or financial relationships that could be construed as a potential conflict of interest.

## Abbreviations

ALS: amyotrophic lateral sclerosis
CNV: copy number variation
dPCR: digital polymerase chain reaction
FUS: fused in sarcoma
GTF2H2: general transcription factor IIH
LMN: lower motor neuron
MLPA: multiple ligation-dependent probe amplification
NAIP: neuronal apoptosis inhibitory protein
OMIM: Online Mendelian Inheritance in Man
PCR-RFLP: polymerase chain reaction-restriction fragment length polymorphism
PMA: progressive muscular atrophy
SERF1A: small EDRK-rich factor 1A
SMA: spinal muscular atrophy
SMNΔ7: survival motor neuron lacking exon 7
SMN1: survival motor neuron 1
SMN2: survival motor neuron 2
snRNP: small nuclear ribonucleoprotein particle
SOD1: superoxide dismutase 1
TDP-43: TAR DNA binding protein-43kDa
UMN: upper motor neuron.
